# *Programmed death-1* polymorphisms is associated with risk of esophagogastric junction adenocarcinoma in the Chinese Han population: A case-control study involving 2,740 subjects

**DOI:** 10.18632/oncotarget.17338

**Published:** 2017-04-21

**Authors:** Weifeng Tang, Shuchen Chen, Yu Chen, Jihong Lin, Jiangbo Lin, Yafeng Wang, Chao Liu, Mingqiang Kang

**Affiliations:** ^1^ Department of Thoracic Surgery, The Union Clinical Medical College of Fujian Medical University, Fuzhou, Fujian Province, China; ^2^ Department of Medical Oncology, Fujian Provincial Cancer Hospital, Fujian Medical University Cancer Hospital, Fuzhou, Fujian Province, China; ^3^ Department of Cardiology, The People's Hospital of Xishuangbanna Dai Autonomous Prefecture, Jinghong, Yunnan Province, China; ^4^ Department of Cardiothoracic Surgery, Affiliated People's Hospital of Jiangsu University, Zhenjiang, Jiangsu Province, China; ^5^ Key Laboratory of Ministry of Education for Gastrointestinal Cancer, Fujian Medical University, Fuzhou, Fujian Province, China; ^6^ Fujian Key Laboratory of Tumor Microbiology, Fujian Medical University, Fuzhou, Fujian Province, China

**Keywords:** PD-1, polymorphism, esophagogastric junction adenocarcinoma, immune

## Abstract

Single nucleotide polymorphisms (SNPs) in *Programmed cell death 1* (*PD-1*) gene may contribute to the development of cancer. In this study, we selected *PD-1* rs10204525 T>C, rs2227982 A>G, rs36084323 T>C and rs7421861 A>G polymorphisms and designed a hospital-based case-control study to determine the potential relationship between these functional SNPs in *PD-1* gene and esophagogastric junction adenocarcinoma (EGJA) risk. A total of 1,063 EGJA patients and 1,677 controls were enrolled from Eastern Chinese Han population. SNPscan^TM^genotyping assay was used to analyze the genotyping of *PD-1* polymorphisms. We found that *PD-1* rs7421861 A>G polymorphism was associated with the development of EGJA. However, *PD-1* rs2227982 A>G polymorphism was a protective factor for EGJA. In addition, *PD-1* rs36084323 CC homozygote genotype might be associated with a borderline decreased risk of EGJA. In a subgroup analysis, a decreased risk of EGJA in never drinking and never smoking groups was identified. Haplotype comparison analysis suggested that *PD-1* T_rs10204525_G_rs2227982_C_36084323_A_rs7421861_ haplotype significantly decreased the risk of EGJA. However, T_rs10204525_G_rs2227982_C_36084323_G_rs7421861_ haplotype in PD-1 gene may confer risk to EGJA. In conclusion, our study highlights rs2227982 A>G, rs36084323 T>C and rs7421861 A>G polymorphisms and haplotypes in *PD-1* gene, especially within the intron region, are significantly associated with the risk of EGJA. Further case-control studies with larger sample size and detailed gene-environmental data to replicate these findings in different populations are needed to validate our conclusion.

## INTRODUCTION

A steady decline of gastric carcinoma (GC) incidence has been observed worldwide, primarily as a result of a reduction in distal GC [1]. However, GC is the fourth most common malignancy and is a relatively higher incidence in Eastern Asian (e.g. China, Korea and Japan). Esophagogastric junction adenocarcinoma (EGJA) is one of the most rapidly increasing malignancies in North America and Europe and is thought to have different etiology compared to distal GC [2]. Recently, the increasing incidence of EGJA was also identified in Eastern Asian [3, 4]. EGJA is a highly fatal form of malignancies and is a major public health problem in China. The potential risk factors contributing to EGJA are foods preserved by salting, smoking and obesity *et al*. In addition, it is reported that individual's genetic background also plays an important role in pathogenesis of EGJA. Although a number of studies have focused on the etiology of EGJA, it is not well understood. Of late, some studies reported the immune system might be implicated in the etiology of EGJA [5, 6].

*Programmed cell death 1* (*PD-1*) gene was found by Ishida Y in 1992 [7]. It is classified as a member of the immunoglobulin superfamily (IgSF). As other inhibitory costimulatory molecules, PD-1 is expressed on many immunocytes, such as T cells, exhausted T cells, regulatory T cells (Treg), activated monocytes, B cells, natural killer (NK) cells, dendritic cells (DCs) and natural killer T (NKT) cells [8, 9]. PD-1 protein, a transmembrane glycoprotein, consists of an intracellular domain and an extracellular immunoglobulin V domain. PD-1 protein binds two ligands, programmed death ligand-1 (PD-L1) and programmed death ligand-2 (PD-L2). Under common physiological conditions, PD-1 interacts with PD-L1 and PD-L2, and then regulates a immune checkpoint. PD-1 may play a very important role in reducing the function of immune system by inhibiting T-cells and up-regulating Treg [10]. Finally, it decreases autoimmunity and results in self-tolerance.

Most recent studies reported several polymorphisms in *PD-1* gene may be associated with susceptibility to some human autoimmune diseases (e.g., systemic lupus erythematosus, rheumatoid arthritis, type 1 diabetes mellitus, and ankylosing spondylitis *et al*.) [11–16]. Interestingly, accumulating evidences showed that *PD-1* single nucleotide polymorphisms (SNPs) were also correlated with susceptibility to human malignancy (e.g., thyroid cancer, breast cancer, cervical cancer, non-small cell lung cancer and gastric cancer *et al*.) [17–21]. The immune response may be differ greatly among individual tumor hosts, and the potential mechanisms remain unknown. Genetic variations can influence the function of genes and alter the disease phenotypes. Thus, the effect of such functional polymorphisms in immune response genes on cancer risk has attracted our interest. Exploring the relationship of *PD-1* SNPs with EGJA risk may be beneficial for providing prevention and personalized diagnosis. Here, we selected *PD-1* rs10204525 T>C, rs2227982 A>G, rs36084323 T>C and rs7421861 A>G polymorphisms and designed a hospital-based case-control study to determine the potential relationship between these functional SNPs in *PD-1* gene and EGJA risk.

## RESULTS

### Baseline characteristics

The demographics (age and sex) and major risk factors (smoking and drinking status) of participants are summarized in Table 1. The mean ± SD of age was not significant in the EGJA patients compared with non-cancer controls (*P* > 0.05). Our study was well-matched by age and gender. Significant difference was observed on smoking status and alcohol consumption between the EGJA patients and the controls (*P* < 0.001). For *PD-1* rs10204525 T>C, rs2227982 A>G, rs36084323 T>C and rs7421861 A>G polymorphisms, the success rate of genotyping was more than 99%, respectively (Table 2). The minor allele frequency (MAF) in our controls was similar to the data of Chinese population. The distribution of genotype frequencies in controls accorded with Hardy–Weinberg equilibrium (HWE) (Table 2).

**Table 1:** Distribution of selected demographic variables and risk factors in EGJA cases and controls

Variable	Overall Cases (*n* = 1,063)		Overall Controls (*n* = 1,677)		*P*^a^
	*n*	%	*n*	%	
Age (years)	64.19 (±8.63)		63.91 (±10.22)		0.451
Age (years)					0.165
< 64	494	46.47	825	49.19	
≥64	569	53.53	852	50.81	
Sex					0.909
Male	759	71.40	1194	71.20	
Female	304	28.60	483	28.80	
Smoking status					< 0.001
Never	773	72.72	1323	78.89	
Ever	290	27.28	354	21.11	
Alcohol use					< 0.001
Never	908	85.42	1507	89.86	
Ever	155	14.58	170	10.14	

^a^ Two-sided *χ*^2^ test and Student *t* test.

**Table 2:** Primary information for *PD-1* polymorphisms (*PD-1* rs10204525 T>C, rs36084323 T>C, rs7421861 A>G and rs2227982 A>G)

Genotyped polymorphisms	rs10204525 T>C (PD1.6)	rs36084323 T>C (PD1.1)	rs7421861 A>G (PD1.7)	rs2227982 A>G (PD1.9)
Chr	2	2	2	2
Position_37	242792321	242801596	242795350	242793433
Region	3′UTR	Promoter	Intron 1	Exon 5
MAF^a^ for Chinese in database	0.302	0.490	0.165	0.488
MAF in our controls (n = 1,677)	0.280	0.496	0.168	0.492
*P* value for HWE^b^ test in our controls	0.888	0.071	0.232	0.309
% Genotyping value	99.01	99.09	99.09	99.09

^a^MAF: minor allele frequency;

^b^HWE: Hardy–Weinberg equilibrium.

### Association of PD-1 rs10204525 T>C, rs2227982 A>G, rs36084323 T>C and rs7421861 A>G polymorphisms with EGJA

The genotype distributions of *PD-1* rs10204525 T>C, rs2227982 A>G, rs36084323 T>C and rs7421861 A>G polymorphisms are summarized in Table 3. The frequencies of *PD-1* rs2227982 AA, AG, and GG genotypes were 26.13%, 52.74% and 21.13% in 1,063 EGJA patients and 26.40%, 48.75%, and 24.85% in 1,677 controls, respectively. When compared with the frequency of *PD-1* rs2227982 AA genotype, a difference in the frequency of *PD-1* rs2227982 GG genotype was found between the EGJA patients and the controls (crude OR = 0.80, 95% CI: 0.64–1.00, *P* = 0.047). When compared with the frequency of *PD-1* rs2227982 AA/AG genotype, there was a difference in the frequency of *PD-1* rs2227982 GG genotype between EGJA patients and the controls (crude OR = 0.81, 95% CI: 0.67–0.98, *P* = 0.026). Adjustment for age, sex, smoking and drinking, there was also difference in recessive genetic model (GG vs. AA/AG: adjusted OR, 0.81; 95% CI, 0.67–0.97; *P* = 0.024; Table 4).

**Table 3:** The frequencies of *PD-1* rs10204525 T>C, rs36084323 T>C, rs7421861 A>G and rs2227982 A>G polymorphisms in esophagogastric junction adenocarcinoma patients and controls

Genotype	Overall EGJA case (*n* = 1,063)		Overall Controls (*n* = 1,677)	
	*n*	%	*n*	%
rs36084323 T>C				
TT	282	27.09	444	26.52
TC	521	50.05	800	47.79
CC	238	22.86	430	25.69
CT+CC	759	72.91	1,230	73.48
TT+CT	803	77.14	1,244	74.31
C allele	997	47.89	1,660	49.58
rs10204525 T>C				
TT	544	52.36	870	51.97
TC	397	38.21	672	40.14
CC	98	9.43	132	7.89
TC+CC	495	47.64	804	48.03
TT+TC	941	90.57	1,542	92.11
C allele	593	28.54	936	27.96
rs7421861 A>G				
AA	642	61.67	1,166	69.65
AG	358	34.39	454	27.12
GG	41	3.94	54	3.23
AG+GG	399	38.33	508	30.35
AA+AG	1,000	96.06	1,620	96.77
G allele	440	21.13	562	16.79
rs2227982 A>G				
AA	272	26.13	442	26.40
GA	549	52.74	816	48.75
GG	220	21.13	416	24.85
GG+GA	769	73.87	1,232	73.60
GA+AA	821	78.87	1,258	75.15
G allele	989	47.50	1,648	49.22

**Table 4:** Overall and stratified analyses of *PD-1* rs10204525 T>C, rs36084323 T>C, rs7421861 A>G and rs2227982 A>G polymorphisms with esophagogastric junction adenocarcinoma

Genotype	Overall (1,063 cases vs. 1,677 controls)			
	Crude OR (95%CI)	*P*	Adjusted ORa (95%CI)	*P*
rs36084323 T>C				
additive model	0.96 (0.80–1.15)	0.642	0.97 (0.81–1.17)	0.766
homozygote model	0.81 (0.66–1.01)	0.061	0.82 (0.66–1.02)	0.074
Dominant model	0.97 (0.82–1.16)	0.746	0.99 (0.83–1.18)	0.888
Recessive model	0.86 (0.72–1.03)	0.097	0.86 (0.71–1.03)	0.097
rs10204525 T>C				
additive model	0.91 (0.77–1.07)	0.246	0.92 (0.78–1.08)	0.294
homozygote model	1.14 (0.86–1.51)	0.359	1.16 (0.87–1.54)	0.310
Dominant model	0.99 (0.84–1.15)	0.845	1.00 (0.85–1.16)	0.956
Recessive model	1.22 (0.93–1.60)	0.160	1.23 (0.94–1.62)	0.139
rs7421861 A>G				
additive model	**1.39 (1.17–1.64)**	**< 0.001**	**1.39 (1.18–1.65)**	**< 0.001**
homozygote model	1.34 (0.88–2.03)	0.173	1.31 (0.86–1.98)	0.214
Dominant model	**1.43 (1.21–1.68)**	**< 0.001**	**1.43 (1.21–1.68)**	**<0.001**
Recessive model	1.23 (0.81–1.86)	0.327	1.20 (0.79–1.82)	0.394
rs2227982 A>G				
additive model	1.02 (0.85–1.22)	0.845	1.04 (0.86–1.25)	0.690
homozygote model	**0.80 (0.64–1.00)**	**0.047**	0.81 (0.65–1.01)	0.057
Dominant model	1.01 (0.85–1.21)	0.874	1.03 (0.87–1.23)	0.716
Recessive model	**0.81 (0.67–0.98)**	**0.026**	**0.81 (0.67–0.97)**	**0.024**

^a^Adjusted for age, sex, smoking status and alcohol use in a logistic regression model.

The frequencies of *PD-1* rs7421861 AA, AG, and GG genotypes were 61.67%, 34.39% and 3.94% in 1,063 EGJA patients and 69.65%, 27.12%, and 3.23% in 1,677 controls, respectively. When compared with the frequency of *PD-1* rs7421861 AA genotype, there was difference in the frequency of *PD-1* rs7421861 AG genotype between the EGJA patients and the controls (crude OR = 1.39, 95% CI: 1.17–1.64, *P* < 0.001). When compared with the frequency of *PD-1* rs7421861 AA genotype, there was also difference in the frequency of *PD-1* rs7421861 AG/GG genotype between the EGJA patients and the controls (crude OR = 1.43, 95% CI: 1.21–1.68, *P* < 0.001). Adjustments for age, sex, smoking and drinking, the observed results were not essentially changed (AG vs. AA: adjusted OR, 1.39; 95% CI, 1.18–1.65; *P* < 0.001; AG/GG vs. AA: adjusted OR, 1.43; 95% CI, 1.21–1.68; *P* < 0.001; Table 4).

The *PD-1* rs36084323 T>C polymorphism conferred a borderline statistically decreased risk to EGJA in homozygote genetic model (crude OR = 0.81, 95% CI = 0.66–1.01, *P* = 0.061) and recessive genetic model (crude OR = 0.86, 95% CI = 0.72–1.03, *P* = 0.097). When adjusted for age, sex, smoking and drinking, a borderline statistically decreased risk of EGJA was also found in homozygote genetic model (crude OR = 0.82, 95% CI = 0.66–1.02, *P* = 0.074) and recessive genetic model (crude OR = 0.86, 95% CI = 0.71–1.03, *P* = 0.097). However, there was no difference in genotype distribution of *PD-1* rs10204525 T>C polymorphism among EGJA patients and the controls (Table 4).

We used the Power and Sample Size Calculator (http://biostat.mc.vanderbilt.edu/twiki/bin/view/Main/PowerSampleSize) to calculate the power value (α = 0.05) [22]. For *PD-1* rs7421861 A>G, the power value was 0.967 in the additive model and 0.990 in the dominant model. For *PD-1* rs2227982 A>G, the power value was 0.675 in the homozygote model and 0.607 in the recessive model.

### Association of PD-1 rs10204525 T>C, rs2227982 A>G, rs36084323 T>C and rs7421861 A>G polymorphisms with EGJA in different stratification groups

Table 5 summarizes the genotype frequencies of *PD-1* rs10204525 T>C polymorphism in the stratified analysis by gender, age, alcohol consumption and smoking status. There was no significant difference in genotype distribution of *PD-1* rs10204525 T>C polymorphism among EGJA patients and the controls in any subgroup.

**Table 5:** Stratified analyses between PD-1 rs10204525 T>C polymorphism and EGJA risk by sex, age, smoking status and alcohol consumption

Variable	PD-1 rs10204525 T>C (case/control) a			Adjusted OR b (95% CI); *P*				
	TT	TC	CC	TT	TC	CC	TC / CC	CC vs. (TC/TT)
Sex								
Male	384/621	289/476	71/94	1.00	0.96 (0.79–1.17); *P*: 0.704	1.20 (0.86–1.67); *P*: 0.294	1.04 (0.86–1.25); *P*: 0.700	1.24(0.90–1.72); *P*: 0.194
Female	160/249	108/196	27/38	1.00	0.79(0.58–1.08); *P*: 0.140	1.08 (0.63–1.85); *P*: 0.776	0.89(0.66–1.19); *P*: 0.422	1.23 (0.73–2.08); *P*: 0.433
Age								
<64	245/442	187/310	50/71	1.00	1.04 (0.82–1.33); *P*: 0.725	1.26 (0.85–1.87); *P*: 0.260	1.13(0.90–1.42); *P*: 0.283	1.27 (0.86–1.86); *P*: 0.226
≥64	299/428	210/362	48/61	1.00	0.80 (0.64–1.01); *P*: 0.055	1.10 (0.73–1.66); *P*: 0.640	0.88 (0.71–1.09); *P*: 0.237	1.24 (0.83–1.84); *P*: 0.291
Smoking status								
Never	385/679	300/534	68/108	1.00	0.94(0.78–1.13); *P*: 0.505	1.08(0.78–1.50); *P*: 0.644	1.01 (0.84–1.21); *P*: 0.926	1.14 (0.83–1.57); *P*: 0.419
Ever	159/191	97/138	30/24	1.00	0.84 (0.60–1.18); *P*: 0.321	1.60 (0.89–2.88); *P*: 0.115	0.97 (0.71–1.33); *P*: 0.849	1.74 (0.98–3.08); *P*: 0.058
Alcohol consumption								
Never	464/774	339/610	83/121	1.00	0.89(0.74–1.05); *P*: 0.171	1.11 (0.82–1.50); *P*: 0.508	0.96 (0.82–1.14); *P*: 0.660	1.20(0.89–1.61); *P*: 0.230
Ever	80/96	58/62	15/11	1.00	1.11 (0.68–1.79); *P*: 0.680	2.22(0.91–5.43); *P*: 0.080	1.26 (0.80–1.99); *P*: 0.328	2.14 (0.89–5.10); *P*: 0.088

^a^The genotyping was successful in 1063 (97.74%) EGJA cases, and 1677 (99.82%) controls for *PD-1* rs10204525 T>C;

^b^Adjusted for age, sex, smoking status and alcohol consumption (besides stratified factors accordingly) in a logistic regression model.

The genotype frequencies of *PD-1* rs36084323 T>C polymorphism in the stratified analysis by gender, age, alcohol consumption and smoking status are summarized in Table 6. In never smoking group, after adjustment for gender, age, alcohol consumption and smoking status by logistic regression analysis, the *PD-1* rs36084323 CC genotype was associated with a significantly decreased risk of EGJA compared with the TC/TT genotype [CC vs. TC/TT: adjusted OR = 0.80, 95% CI 0.65–0.99, *P* = 0.043 (Table 6)]. In never drink group, after logistic regression analysis, the CC genotype of *PD-1* rs36084323 T>C polymorphism was also associated with a significantly decreased risk of EGJA compared with the TT genotype [CC vs. TT: adjusted OR = 0.78, 95% CI 0.62–0.99, *P* = 0.037 (Table 6)].

**Table 6:** Stratified analyses between PD-1 rs36084323 T>C polymorphism and EGJA risk by sex, age, smoking status and alcohol consumption

Variable	PD-1 rs36084323 T>C (case/control)^a^			Adjusted OR b (95% CI); *P*				
	TT	TC	CC	TT	TC	CC	TC / CC	CC vs. (TC/TT)
Sex								
Male	198/316	368/561	180/314	1.00	1.01 (0.81–1.26); *P*: 0.918	0.88 (0.68–1.14); *P*: 0.331	1.02 (0.83–1.26); *P*: 0.840	0.89(0.72–1.11); *P*: 0.296
Female	84/128	153/239	58/116	1.00	0.88(0.63–1.24); *P*: 0.462	0.69 (0.45–1.04); *P*: 0.077	0.91(0.65–1.26); *P*: 0.561	0.77 (0.54–1.11); *P*: 0.160
Age								
<64	130/215	240/411	112/197	1.00	0.90 (0.69–1.17); *P*: 0.415	0.87 (0.63–1.19); *P*: 0.382	0.96(0.74–1.24); *P*: 0.768	0.96 (0.74–1.26); *P*: 0.769
≥64	152/229	281/389	126/233	1.00	1.03 (0.80–1.33); *P*: 0.833	0.79 (0.58–1.05); *P*: 0.098	0.99 (0.78–1.27); *P*: 0.962	0.78 (0.61–1.00); *P*: 0.053
Smoking status								
Never	195/352	395/631	164/338	1.00	1.04(0.84–1.28); *P*: 0.750	0.80(0.62–1.03); *P*: 0.078	1.04 (0.85–1.27); *P*: 0.715	0.80 (0.65–0.99); P: 0.043
Ever	87/92	126/169	74/92	1.00	0.80(0.55–1.17); *P*: 0.244	0.90 (0.58–1.38); *P*: 0.615	0.85 (0.60–1.22); *P*: 0.379	1.04 (0.72–1.49); *P*: 0.834
Alcohol consumption								
Never	235/389	450/722	202/394	1.00	0.95(0.78–1.16); *P*: 0.638	0.78 (0.62–0.99); P: 0.037	0.97 (0.80–1.17); *P*: 0.739	0.83(0.68–1.01); *P*: 0.058
Ever	47/55	71/78	36/36	1.00	1.10 (0.65–1.86); *P*: 0.712	1.18(0.63–2.21); *P*: 0.598	1.13(0.69–1.84); *P*: 0.630	1.11 (0.65–1.92); *P*: 0.697

^a^The genotyping was successful in 1063 (97.93%) EGJA cases, and 1677 (99.82%) controls for *PD-1* rs36084323 T>C;

^b^Adjusted for age, sex, smoking status and alcohol consumption (besides stratified factors accordingly) in a logistic regression model.

In the stratified analysis by gender, age, alcohol consumption and smoking status, the genotype frequencies of *PD-*1 rs7421861 A>G polymorphism are summarized in Table 7. After adjustment by logistic regression analysis, we found that *PD-*1 rs7421861 A>G polymorphism was associated with a significantly increased risk of EGJA in some subgroups [male group: AG vs. AA: adjusted OR = 1.40, 95% CI 1.15–1.70, *P* = 0.001 and AG/GG vs. AA: adjusted OR = 1.41, 95% CI 1.16–1.71, *P* = 0.001; female group: AG/GG vs. AA: adjusted OR = 1.46, 95% CI 1.07–2.00, *P* = 0.017; <64 years subgroup: AG vs. AA: adjusted OR = 1.62, 95% CI 1.27–2.08, *P* < 0.001; GG vs. AA: adjusted OR = 2.07, 95% CI 1.12–3.83, *P* = 0.021 and AG/GG vs. AA: adjusted OR = 1.73, 95% CI 1.36–2.20, *P* < 0.001; never smoking group: AG vs. AA: adjusted OR = 1.42, 95% CI 1.17–1.73, *P* < 0.001 and AG/GG vs. AA: adjusted OR = 1.45, 95% CI 1.20–1.75, *P* < 0.001 and never drinking group: AG vs. AA: adjusted OR = 1.42, 95% CI 1.19–1.70, *P* < 0.001 and AG/GG vs. AA: adjusted OR = 1.46, 95% CI 1.22–1.73, *P* < 0.001 (Table 7)].

**Table 7:** Stratified analyses between *PD-1* rs7421861 A>G polymorphism and EGJA risk by sex, age, smoking status and alcohol consumption

Variable	PD-1 rs7421861 A>G (case/control)^a^			Adjusted OR b (95% CI); *P*				
	AA	AG	GG	AA	AG	GG	AG/GG	GG vs. (AG/AA)
Sex								
Male	455/819	262/331	29/41	1.00	1.40 (1.15–1.70); P: 0.001	1.20 (0.74–1.97); *P*: 0.462	1.41 (1.16–1.71); P: 0.001	1.10 (0.67–1.79); *P*: 0.712
Female	187/347	96/123	12/13	1.00	1.37 (0.99–1.89); *P*: 0.058	1.65 (0.73–3.69); *P*: 0.228	1.46 (1.07–2.00); P: 0.017	1.55(0.69–3.46); *P*: 0.286
Age								
<64	286/590	173/212	23/21	1.00	1.62 (1.27–2.08); P: < 0.001	2.07 (1.12–3.83); P: 0.021	1.73 (1.36–2.20); P: < 0.001	1.82 (0.99–3.35); *P*: 0.054
≥64	356/576	185/242	18/33	1.00	1.21 (0.96–1.53); *P*: 0.108	0.87 (0.48–1.56); *P*: 0.633	1.20 (0.96–1.50); *P*: 0.113	0.83 (0.46–1.49); *P*: 0.531
Smoking status								
Never	465/925	263/353	26/43	1.00	1.42 (1.17–1.73); P: < 0.001	1.16 (0.70–1.92); *P*: 0.558	1.45 (1.20–1.75); P: < 0.001	1.07 (0.65–1.75); *P*: 0.804
Ever	177/241	95/101	15/11	1.00	1.29 (0.91–1.82); *P*: 0.149	1.85 (0.82–4.17); *P*: 0.139	1.36 (0.98–1.90); *P*: 0.068	1.72 (0.77–3.85); *P*: 0.188
Alcohol consumption								
Never	544/1050	311/408	32/47	1.00	1.42 (1.19–1.70); P: < 0.001	1.26 (0.80–2.00); *P*: 0.326	1.46 (1.22–1.73); P: < 0.001	1.15 (0.73–1.82); *P*: 0.541
Ever	98/116	47/46	9/7	1.00	1.14 (0.69–1.89); *P*: 0.609	1.73 (0.59–5.09); *P*: 0.322	1.21 (0.75–1.95); *P*: 0.441	1.66 (0.57–4.83); *P*: 0.356

^a^ The genotyping was successful in 1063 (97.93%) EGJA cases, and 1677 (99.82%) controls for *PD-1* rs7421861 A>G;

^b^ Adjusted for age, sex, smoking status and alcohol consumption (besides stratified factors accordingly) in a logistic regression model.

In the stratified analysis by gender, age, alcohol consumption and smoking status, the genotype frequencies of *PD-1* rs2227982 A>G polymorphism are summarized in Table 8. After adjustment by logistic regression analysis, the *PD-1* rs2227982 GG genotype was associated with a decreased risk of EGJA compared with the AG/AA genotype in three groups [≥ 64 years subgroup: GG vs. AG/AA: adjusted OR = 0.72, 95% CI 0.56–0.93, *P* = 0.011; never smoking group: GG vs. AG/AA: adjusted OR = 0.74, 95% CI 0.60–0.92, *P* = 0.008 and never drinking group: GG vs. AG/AA: adjusted OR = 0.76, 95% CI 0.62–0.93, *P* = 0.008 (Table 8)]. Additionally, compared with the *PD-1* rs2227982 AA genotype, the *PD-1* rs2227982 GG genotypes were also associated with a significantly decreased risk of EGJA in never drink group [GG vs. AA: adjusted OR = 0.76, 95% CI 0.60–0.97, *P* = 0.024 (Table 8)].

**Table 8:** Stratified analyses between *PD-1* rs2227982 A>G polymorphism and EGJA risk by sex, age, smoking status and alcohol consumption

Variable	PD-1 rs2227982 A>G (case/control) a			Adjusted OR b (95% CI); *P*				
	AA	AG	GG	AA	AG	GG	AG/GG	GG vs. (AG/AA)
Sex								
Male	188/315	393/572	165/304	1.00	1.12(0.89–1.39); *P*: 0.333	0.87 (0.67–1.14); *P*: 0.310	1.10 (0.89–1.35); *P*: 0.399	0.83 (0.67–1.03); *P*: 0.093
Female	84/127	156/244	55/112	1.00	0.87 (0.62–1.23); *P*: 0.433	0.66 (0.43–1.01); *P*: 0.057	0.90 (0.65–1.24); *P*: 0.513	0.75 (0.52–1.08); *P*: 0.124
Age								
<64	126/212	253/426	103/185	1.00	0.93 (0.71–1.22); *P*: 0.597	0.87 (0.63–1.20); *P*: 0.383	0.99 (0.77–1.29); *P*: 0.948	0.93 (0.71–1.23); *P*: 0.625
≥64	146/230	296/390	117/231	1.00	1.13(0.87–1.45); *P*: 0.359	0.76 (0.56–1.03); *P*: 0.074	1.06 (0.83–1.35); *P*: 0.666	**0.72 (0.56–0.93); P: 0.011**
Smoking status								
Never	188/351	415/638	151/332	1.00	1.11 (0.90–1.37); *P*: 0.339	0.77 (0.60–1.00); *P*: 0.051	1.09 (0.89–1.34); *P*: 0.422	**0.74(0.60–0.92); P: 0.008**
Ever	84/91	134/178	69/84	1.00	0.84 (0.58–1.22); *P*: 0.365	0.94 (0.61–1.46); *P*: 0.788	0.90 (0.63–1.28); *P*: 0.542	1.06 (0.73–1.54); *P*: 0.752
Alcohol consumption								
Never	224/387	481/739	182/379	1.00	1.04 (0.85–1.26); *P*: 0.714	**0.76 (0.60–0.97); P: 0.024**	1.03 (0.85–1.24); *P*: 0.783	**0.76 (0.62–0.93); P: 0.008**
Ever	48/55	68/77	38/37	1.00	0.99 (0.59–1.67); *P*: 0.961	1.30 (0.70–2.41); *P*: 0.410	1.08 (0.67–1.76); *P*: 0.753	1.30 (0.76–2.24); *P*: 0.339

^a^The genotyping was successful in 1063 (97.93%) EGJA cases, and 1677 (99.82%) controls for *PD-1* rs2227982 A>G;

^b^Adjusted for age, sex, smoking status and alcohol consumption (besides stratified factors accordingly) in a logistic regression model.

### SNP haplotypes

Using SHESIS software (http://analysis.bio-x.cn/myAnalysis.php) [23], we constructed six haplotypes (Table 9). We found that TGCA haplotypes with the order of *PD-1* rs10204525 T>C, rs2227982 A>G, rs36084323 T>C and rs7421861 A>G polymorphisms in gene position significantly decreased the risk of EGJA (OR = 0.83, 95% CI = 0.71–0.96; *P* = 0.015). However, TGCG and other haplotypes with the same order of *PD-1* SNPs in gene position significantly increased the risk of EGJA (OR = 22.19, 95% CI = 5.27–93.56; *P* < 0.001 and OR = 2.50, 95% CI = 1.73–3.60; *P* < 0.001).

**Table 9:** PD-1 haplotype frequencies (%) in cases and controls and risk of esophagogastric junction adenocarcinom

Haplotypes	Case (n=2,126)		Control (n=3,354)		Crude OR (95% CI)	*P*
	*n*	%	*n*	%		
T_rs10204525_A_rs2227982_T_rs36084323_A_rs74218_61	1000	49.24	1644	49.07	1.00	
C_rs10204525_G_rs2227982_C_rs36084323_G_rs7421861_	354	17.43	544	16.24	1.07(0.92–1.25)	0.394
T_rs10204525_G_rs2227982_C_rs36084323_A_rs7421861_	347	17.09	688	20.54	**0.83(0.71–0.96)**	**0.015**
C_rs10204525_G_rs2227982_C_rs36084323_A_rs7421861_	198	9.75	374	11.16	0.87(0.72–1.05)	0.150
T_rs10204525_A_rs2227982_C_rs36084323_A_rs7421861_	29	1.43	48	1.43	0.99(0.62–1.59)	0.977
T_rs10204525_G_rs2227982_C_rs36084323_G_rs7421861_	27	1.33	2	0.06	**22.19(5.27–93.56)**	**<0.001**
Others	76	3.74	50	1.49	**2.50(1.73–3.60)**	**<0.001**

## DISCUSSION

EGJA is considered as a separated carcinoma entirety of upper digestive tract malignancies [24]. Although the incidence of GC is declining, the incidence of EGJA rapidly increases in both western (e.g. North America and Europe) and eastern Asian countries [2–4, 25]. Thus, EGJA is one of the most prevalent malignancies worldwide. The etiology of EGJA is very complicated. Accumulating evidences demonstrated that SNPs in some immune response genes might be associated with risk of cancer [6, 26–28]. In consideration of the vital role of PD-1 in tumor immunology, we chose *PD-1* rs10204525 T>C, rs2227982 A>G, rs36084323 T>C and rs7421861 A>G polymorphisms to examine their potential roles in EGJA. In the present study, we found that *PD-1* rs2227982 A>G and rs36084323 T>C polymorphisms might decrease the risk of EGJA. However, *PD-1* rs7421861 A>G might be a risk factor for EGJA. In addition, we found T_rs10204525_G_rs2227982_C_rs36084323_A_rs7421861_ haplotypes significantly decreased the risk of EGJA. On the contrary, T_rs10204525_G_rs2227982_C_rs36084323_G_rs7421861_ and other haplotypes increased the risk of EGJA.

*PD-1* rs7421861 A>G polymorphism locates on intron 1, where a lot of regulatory components and splicing control elements may interact with it [29]. Some epidemiological studies focused on the effect of *PD-1* rs7421861 A>G locus on the development of multiple cancers; however, the results remained inconsistent. Several case-control reported that *PD-1* rs7421861 A>G polymorphism was not associated with cancer risk [19, 27, 28, 30]. However, Ge *et al*. found *PD-1* rs7421861 A>G polymorphism might increase the risk of overall colorectal cancer [31]. Recently, a meta-analysis indicated that *PD-1* rs7421861 A>G was correlated with a borderline statistically increased risk of overall cancer [32]. In the present study, we found *PD-1* rs7421861 A>G polymorphism might be associated with the susceptibility of EGJA, which was similar to the findings of the previous studies [31, 32]. This study did not examine the potential effect of this polymorphism on regulating the expression of PD-1 in EGJA patient blood samples. However, in the future, we will conduct a further study to assess whether *PD-1* rs7421861 A>G polymorphism is associated with the inhibited activation of T cells in EGJA patients, which may impede the surveillance mechanism of immune system.

*PD-1* rs2227982 A>G polymorphism, a SNP in Exon 5, encodes a Val to Ala substitution in the extra-cellular domain of PD-1 receptor during protein synthesis, which influences the sequence, and may alter the function of PD-1 protein. In this study, we found that *PD-1* rs2227982 A>G polymorphism was associated with the decreased risk of EGJA risk. Ren *et al*. reported that *PD-1* rs2227982 A>G polymorphism was associated with the development of breast cancer [19]. In addition, several studies reported there was no significant association between *PD-1* rs2227982 A>G polymorphism and cancer (e.g. esophageal squamous cell carcinoma, colorectal cancer, breast cancer and non-small cell lung cancer) [27, 28, 30, 31]. A recent meta-analysis suggested that *PD-1* rs2227982 A>G polymorphism might be not associated with the risk of overall cancer. However, we found that only five case-control studies with relatively small sample sizes were included in this pooled analysis. The current evidence of the relationship might be very limited. Therefore, whether the Val to Ala substitution in the extra-cellular domain of PD-1 recepter does alter biological activity of PD-1 protein is needed to be further assessed.

Previous report showed that *PD-1* rs36084323 CC was associated with a significantly decreased risk of breast cancer compared with the TT genotype [30]. In the present study, we found a borderline statistically decreased risk to EGJA in homozygote genetic model and recessive genetic model. In the stratified analysis by gender, age, alcohol consumption and smoking status, the decreased risk was observed in never smoking group and in never drinking group. To the best of our knowledge, variants in the promoter region of functional gene may influence an initial binding of transcription factors with sequence motifs, and further alter gene expression [33, 34]. Accordingly, as a variant in promoter region of PD-1 gene, *PD-1* rs36084323 CC genotype may also affect the activation of transcription, and then regulate the expression of PD-1 gene and increase the risk of EGJA.

In this study, we constructed haplotypes to explore the potential inherited patterns of haplotype. We found that *PD-1* T_rs10204525_G_rs2227982_C_36084323_A_rs7421861_ haplotype significantly decreased the risk of EGJA. However, T_rs10204525_G_rs2227982_C_36084323_G_rs7421861_ haplotype in *PD-1* gene might significantly increase the risk of EGJA. We first studied the relationship of haplotypes in *PD-1* rs10204525 T>C, rs2227982 A>G, rs36084323 T>C and rs7421861 A>G polymorphisms with EGJA susceptibility. Compared *PD-1* T_rs10204525_G_rs2227982_C_36084323_A_rs7421861_ with T_rs10204525_G_rs2227982_C_36084323_G_rs7421861_ haplotype, we also found that A→G variation in *PD-1* rs7421861 locus might significantly inverse the risk of haplotype to EGJA.

In addition, some limitations in this case-control study should be acknowledged. All participants were enrolled in three hospitals from Eastern Chinese Han population. Although the genotype distributions of *PD-1* rs10204525 T>C, rs2227982 A>G, rs36084323 T>C and rs7421861 A>G polymorphisms in controls were consistent with HWE and the MAF in the selected controls was very close to the data of Chinese (Table 2), the non-cancer controls might not well-represent the whole Chinese population. As well, only four important SNPs (MAF ≥ 0.05) in *PD-1* gene were selected, which might not be enough to determine the total genetic susceptibility in *PD-1* gene. Future, a fine-mapping study with larger sample sizes, multiple centers and detailed risk factors is need to confirm these primary findings.

In summary, our study highlights rs2227982 A>G, rs36084323 T>C and rs7421861 A>G polymorphisms and haplotypes in *PD-1* gene, especially within the intron region, may be associated with the risk of EGJA in Eastern Chinese Han population. Our primary findings suggest that *PD-1* genetic variation may be beneficial for the exploration of Eastern Chinese subjects genetically susceptible to EGJA.

## MATERIALS AND METHODS

### Subjects

A total of 2,740 participants were recruited in this case-control study. Among them, 280 EGJA patients were enrolled from the Affiliated Union Hospital of Fujian Medical University and Fujian Medical University Cancer Hospital from January 2014 to May 2016. In addition, 783 EGJA patients were recruited from the Affiliated People's Hospital of Jiangsu University from January 2008 to November 2016. All cases with histologically confirmed EGJA were enrolled in the present study. EGJA patients who had a history of another malignancy or received prior chemoradiotherapy were excluded. At the same time, 1,677 subjects who underwent health check in these hospitals were recruited as non-cancer controls. Controls were matched with EGJA cases in terms of gender and age. All participants were unrelated Eastern Chinese Han population. Each participant signed the written informed consent. The study was approved by the Review Boards of Jiangsu University (Zhenjiang, China) and Fujian Medical University (Fuzhou, China), in accordance with the Declaration of Helsinki. Two experienced doctors interviewed each individual and collected the relevant risk factors and demographic variables. The corresponding data are summarized in Table 1. Each study participant provided an ethylenediamine tetraacetic acid (EDTA)-anticoagulated intravenous blood sample after an overnight fast. The approved guidelines were used as experimental protocol.

### Selection of SNPs

The tagging polymorphisms among the gene region of *PD-1* (upstream and downstream of gene extending 5 Kb, respectively) were collected from the CHB population via an internet the HapMap Project (http://hapmap.ncbi.nlm.nih.gov/index.html.en) and were conducted with Haploview 4.2 software with the criterion of pairwise linkage disequilibrium (LD) *r*^2^ threshold of 0.8 between polymorphisms (with a minimum LD of *r*^2^ > 0.8) [35]. Finally, SNPs with a HWE *P* ≥ 0.05, MAF ≥ 0.05 and call rate ≥ 95 % in the CHB population were included [36]. *PD-1* rs36084323 T>C polymorphism locates on the promoter of *PD-1* gene, which region may influence the transcription of *PD-1*. Thus, in this study, we also included this important SNP. The primary information of PD-1 functional SNPs is summarized in Table 2.

### DNA extraction and genotyping

Peripheral blood sample was collected and stored at −20°C. Using the Promega Genomic DNA Purification Kit (Promega, Madison, USA), the genomic DNA was carefully extracted from lymphocytes. The obtained genomic DNA was frozen at −80°C. SNPscan^TM^ genotyping assay (Gnensky Biotechologies Inc., Shanghai, China) was used to analyze the genotyping of *PD-1* rs10204525 T>C, rs2227982 A>G, rs36084323 T>C and rs7421861 A>G polymorphisms. For quality control, 110 DNA samples (4%) were randomly selected. The genotypes of *PD-1* rs10204525 T>C, rs2227982 A>G, rs36084323 T>C and rs7421861 A>G polymorphisms were reanalyzed by different laboratory technicians. The reproducibility was 100%. The success rate of *PD-1* genotyping is shown in Table 2.

### Statistical analysis

Age was expressed as the mean ± standard deviation (SD). And we used Student's *t*-test to calculate the differences for continuous variables between EGJA patients and controls. An internet-based calculator (http://ihg.gsf.de/cgi-bin/hw/hwa1.pl) was used to determine the deviation of genotype frequencies from HWE. Chi-square test (*χ*^2^) was conducted to compare the categorical variables (e.g. age, sex, smoking status, and drinking) and the genotype distributions between EGJA patients and non-cancer controls. Multivariate logistic regression was used to obtain the crude/adjusted odds ratios (OR) and their 95 % confidence intervals (CI) for the relationship of *PD-1* rs10204525 T>C, rs2227982 A>G, rs36084323 T>C and rs7421861 A>G polymorphisms with EGJA risk. The SAS software (Version 9.4; SAS Institute Inc., Cary, NC, USA) was used to analyze all data. SHESIS online software [(http://analysis.bio-x.cn/myAnalysis.php), Bio-X Inc., Shanghai, China] was harnessed to construct the haplotypes of *PD-1* gene [23]. The criterion of statistical significance was defined as *P* < 0.05 (two-tailed). We performed the Power and Sample Size Calculator (http://biostat.mc.vanderbilt.edu/twiki/bin/view/Main/PowerSampleSize) to assess the power value of the study (α = 0.05) [22].
